# Design of Phase Gradient Coding Metasurfaces for Broadband Wave Modulating

**DOI:** 10.1038/s41598-018-26981-6

**Published:** 2018-06-06

**Authors:** Yang Zhou, Guori Zhang, Haiyan Chen, Peiheng Zhou, Xin Wang, Linbo Zhang, Li Zhang, Jianliang Xie, Longjiang Deng

**Affiliations:** State Key Laboratory of Electronic Thin Films and Integrated Devices, National Engineering Research Center of Electromagnetic Radiation Control Materials, Key Laboratory of Multi-Spectral Absorbing Materials and Structures of Ministry of Education, University of Electronic Science and Technology of China, Chengdu, 610054 China

## Abstract

Wave modulating is one of the most interesting applications of metasurfaces. It requires an effective method to design metasurfaces with arbitrary space-variant phase. In this paper, we proposed an optimized design method for arbitrarily modulating wave based on the Genetic Algorithm, which is efficient to optimize designated radiation patterns according to application requirements. In order to verify the availability of the method, wave modulating of single lobe radiation at 10 GHz and broadband 3-lobes radiation at X band are optimized. For wave modulating of single lobe radiation, eight basic codes are chosen to excite the specific phases evenly dispersed from 0 to 2π for smooth phase gradient, and the 8 × 8, 20 × 20 and 40 × 40 arrays of the basic codes are optimized. It proves that the wave modulation accuracy is enhancing with the increase of elements quantity. For wave modulating of 3-lobes radiation, the 20 × 20 arrays are proposed and optimized, and their basic codes are increased to 32 for meeting the broadband requirement. Its broadband wave modulating has been verified by simulation and experiment, and it is shown that the directional 3-lobes radiation patterns keeps nearly stable within the broadband frequency range of 8.7–11.3 GHz.

## Introduction

Metasurfaces, as ultrathin planar artificial structures, have attracted much attention in recent years. Many novel applications were realized with such configurations, including polarization control^1–3^, reflection-phase modulation^4–6^, perfect absorption^7–9^, focusing^10–12^, and holograms^13–15^. In microwave bands, wave modulating, including anomalous reflection, refraction and focusing^16–20^, is one of the most interesting phenomena, which can be used in radome, super-lens and radar cross section (RCS) reduction. According to the recent development in this area, the reported metasurfaces can be basically classified into two categories, dynamic^21–23^ and static^11–14^. Dynamic metasurfaces are usually active. Based on FPGA or other logical circuits, the phase gradient becomes tunable and therefore realizes different functions in one configuration. For static metasurfaces, they have the characteristics of ultrathin thickness and relatively simple layout, which can be used in severe environments where the dynamic component is not applicable. The problems of static metasurfaces for arbitrary wave manipulating are the limited operating bandwidth and time-consuming layout design.

For layout design, there are two strategies to solve the problem. One is enhancing the continuity of phase gradient of basic elements. According to the generalized Snell’s law^24^, once the phase gradient *dФ/dx* along the interface of two media is ideally continuous, arbitrary wave modulating can be achieved. However, the phase gradient is actually generated by the phase difference between adjacent subwavelength elements of the metasurface, and the size of elements cannot be infinitely small. Hence, for metasurfaces with finite size, the phase gradient is piecewise rather than continuous. Promoting the continuity of phase gradient is helpful for modulating wave flexibly. The other one is optimizing the layout of metasurfaces. In Cui’s report^25^, radiation of metasurfaces can be expressed by a sequence of ‘0’ and ‘1’ elements, which is named as ‘coding metamaterials’. Essentially, different combinations of ‘0’ and ‘1’ elements can be equivalent to big units with different reflection phases, so that it can generate a desired phase gradient and radiation by reasonable layout. Therefore, it is necessary to effectively and efficiently design the reasonable layout of metasurfaces for achieving arbitrary wave modulating. Genetic Algorithm (GA) is employed due to its efficient global search and optimization, which has been used in Computer Science^26^, Sociology^27^, Biology^28^ and so on. By simulating the process of natural selection, GA is able to obtain the optimization with global search and convergence^29^.

For bandwidth expansion, the phase gradient of basic elements should keep constant in operating bandwidth. However, for static metasurfaces, phase gradient is normally varying with the work frequency, which leads to the difference of wave modulating. Broadband wave modulating can be design by optimization algorithm, which reduces the difference in between the radiations of different work frequencies. Compared to the design of broadband RCS reduction^9,30^, the design of broadband wave modulating is more difficult and rarely studied.

In this paper, we proposed an optimized design method for arbitrary modulating wave based on the Genetic Algorithm. Compare to the design of RCS reduction^30,31^, the random distribution metasurfaces have been designed and fabricated to modulate the radiation direction. Different from the typical 0–1 coding metasurfaces^25,30–32^, the multi-coding metasurfaces are able to draw into various phase gradient. The multi-coding elements are composed of different size metal patterns, with reflection phases distributed evenly from 0 to 2π. It will improve the flexibility of wave manipulation based on generalized Snell’s law^24^. In addition, the directivities of metasurfaces have been calculated to measure the direction and magnitude of wave manipulation. The metasurfaces of 8 × 8, 20 × 20 and 40 × 40 arrays have been simulated. According to the comparison of different size arrays, it is demonstrated that the directivity is enhancing with the increase of elements quantity. In order to verify the performance of broadband wave modulating, the basic elements of the phase gradient metasurfaces further increase to 32, and optimally arranged into a 20 × 20 metasurfaces array. It concludes that the Radar Cross Section (RCS) keeps stable from 8.7 GHz to 11.3 GHz, which float in a 3 dB range.

## Design Method

An M × N array of elements of varied reflection phase under plane-wave normal incidence is considered. According to array theory^31^, the far-field function is described as

1 $$\begin{array}{rcl}f({\rm{\theta }},{\rm{\phi }}) & = & \sum _{m}\sum _{n}({f}_{i}\cdot {f}_{r}\cdot {f}_{p})\\ {f}_{r}(m,n) & = & \exp [-i\phi (m,n)]\\ {f}_{p}(m,n) & = & \exp \{-ikDsin\theta [cos\phi (m-\frac{1}{2})+sin\phi (n-\frac{1}{2})]\}\end{array}$$

where *θ* and *φ* are the elevation and azimuth angles of an arbitrary direction, *k* is the wave vector in free space, *D* is the size of elements, *f*_*r*_ and *f*_*p*_ represent the phase components of each element attributed to reflection and position respectively. Note that *f*_*i*_ is the phase component of incident wave, which can be seen as 1 for each lattice due to the normal incidence of plane-wave in this paper.

**Figure 1:**
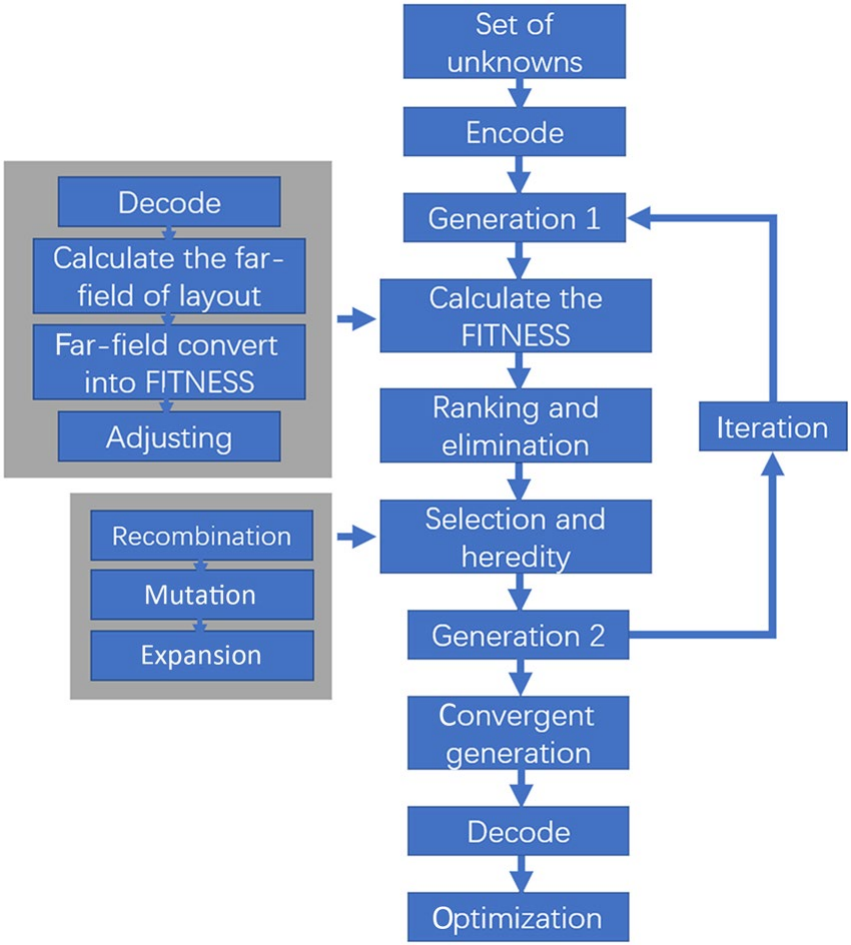
Flowchart of the optimization algorithm. The GA is employed to design the optimal layout of metasurfaces.

In the case of metasurfaces layout optimization, each element can be regarded as a variable contributing to the total radiation. Total radiation of a determined layout can be calculated through expression 1, but it is difficult to use the local optimization methods to obtain the best layout according to a desired radiation. Compared to the Newton method, gradient method and other determined local methods, global optimization algorithm is good at solving the multi-variable and multi-constraint complex model. It can obtain approximate optimal solution for engineering application in the short time by randomly global searching. GA is a global optimization algorithm that simulates the natural selection process. The solving process of GA is independent of the problem and has no requirement for search space, such as function derivability and continuity. In addition, GA directly operates on the coding sequences of the parameter set, which could be consist of graphs, trees and other abstract objectives. Therefore, GA is able to optimize the metasurfaces layout according to the target. Similar to the law of evolution, all the unknowns are encoded into a binary sequence of gene. The transition function (TF) translates every gene into an individual, such as Eq. 1. A number of individuals form a generation. By evaluating the fitness measured by the objective function (OF), the individuals of a generation are ranked and some of the poorer ones will be eliminated. Afterwards, by gene copy, recombination and mutation, the newer individuals are generated and forms the next generation. The process of copy and recombination develop the advantage of individuals, and the mutation make the optimization avoiding local convergence. The fitness has no change more than 50 generations and the quantity of last change is smaller than the 0.01% of the current fitness, then we assume the fitness reaches convergence. In the case of wave modulating, the layout parameters of metasurfaces are the unknowns. The radiation represents the individual and the far-field function in Eq. 1 is the TF. The expectancy of radiation pattern is the OF, which can be expressed as

2 $$OF=\,{\rm{\max }}({\sum }_{i=1}^{N}{\alpha }_{i}\cdot f({\theta }_{i},{\phi }_{i}))$$

where *θ*_*i*_ and *φ*_*i*_ represent the *i*-th far field radiation direction that needs to be manipulated, and *α*_*i*_ is the weight coefficient of each expectancies. Then, the problem of wave manipulating is transformed to solving the maximum of Eq. 2. Figure 1 shows the flow chart of the proposed method for optimizing the layout.

## Optimizing Design of Single Lobe Radiation

**Figure 2:**
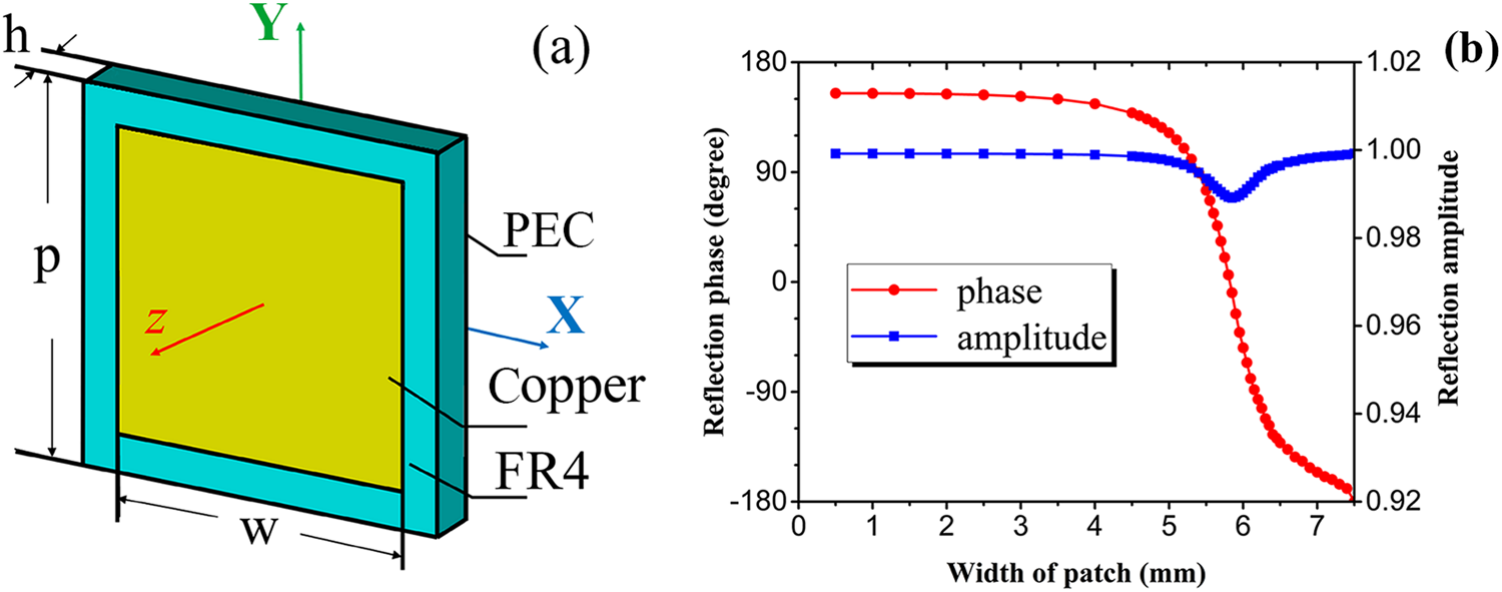
Schematic of element and its reflection properties. (**a**) The copper patch has a conductivity of 5.8 × 10^7^ S/m with a width of *w*; The FR4 substrate backed by perfect electric conductor (PEC) plane has a thickness of *h* = 1 mm with a dielectric constant of 4.6 and loss tangent of 0.001, and the periodicity of the element is *p* = 7.5 mm. (**b**) Simulated reflection phase and amplitude at 10 GHz for the element with varying patch width is obtained by Finite Integral Technology (FIT).

In order to verify the optimization method for wave manipulating, we choose metallic square patches as unit cell pattern. The element illustrated by Fig. 2a is a typical sandwich configuration with FR4 substrate. Eight elements numbered from ‘1’ to ‘8’ own different reflection phases by varying the patch width *w*. Table 1 shows the detailed geometrical parameters of each element. Due to the metal ground on the back, the entire structure is perfectly reflective for incident waves. The reflection amplitude remains above 0.98 and the reflection phase changes from −180° to 155° with varying *w*, as shown in Fig. 2b. Resonance happens near *w* = 6 mm where the phase changes rapidly. Hence, the widths of some elements are chosen very closely to have their reflection phase changing evenly from 0 to 2π. Considering a simple wave manipulating goal of single lobe radiation at *θ*_1_ = 30° and *φ*_1_ = 30° for normal incidence, a metasurface of 8 × 8 array is firstly optimized with the GA.

**Table 1:** The reflection phase of eight elements.

No.	1	2	3	4	5	6	7	8
width	7.5	6.55	6.15	5.95	5.8	5.65	5.4	4.65
phase	−180	−135.0	−88.2	−41.3	5.8	46.0	89.5	135.5

**Figure 3:**
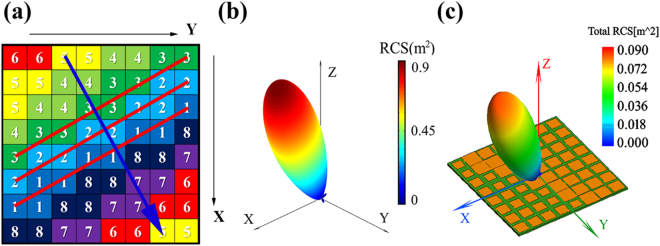
The layout of the optimal metasurface, the calculation and simulation of far field performance under normal incidence at 10 GHz. (**a**) the layout has been optimized with GA, where the different numbers represent elements of different patch width. The red line shows the distribution of elements owing the same reflection phase and the blue arrow shows the direction of phase gradient. (**b**) The calculation of far filed is obtained by the TF and the radiation is *θ*_1_ = 30° and *φ*_1_ = 30°. (**c**) The simulation of far filed is obtained by multilevel fast multipole algorithm (MLFMA), which also shows the radiation *θ*_1_ = 30° and *φ*_1_ = 30°.

The layout of metasurfaces can be encoded into 3 × 8 × 8-bit binary sequence, where every 3-bit binary sequence can represent the eight elements of metasurfaces, then multiplied by 8 × 8 array, the 192-bit binary sequence can depict all the layout of metasurfaces. For each iteration of GA optimization, new individuals are created through one-point crossover recombination and mutation, which replace 10% poor performance individuals to form a new generation. The optimization speed is determined by the sequence length and the number of iterations that reach convergence. With the help of CPU Inter X5680, it takes 30 seconds to reach convergence after 360 generation for optimizing 8 × 8 array. The layout has a regular distribution on surface, which is shown in Fig. 3a and marked by red line and blue arrow. Obviously, the regularity looks simply that satisfied with the generalized Snell’s law. It can be estimated that the angle of the red line to y axis and blue arrow to x axis are close to 30°. The red line represents the distribution of elements that owing the same reflection phase, and dx is determined by the distance between adjacent red lines. Therefore, the phase gradient dФ/dx = 45°/(p/sin30°), and θ_1_ = 30° can be calculated according to the generalized Snell’s law. In addition, the blue arrow is the direction of phase gradient, which shows the azimuth of radiation φ_1_ = 30°. The results demonstrate the validity and efficiency of optimized design method based on GA. The calculated and simulated radiation patterns of the designed metasurface are shown in Fig. 3b,c. Note that the calculation meets well with the simulation, which confirms the wave modulating ability of the optimal design.

According to the RCS theory^33^, the directivity of metasurfaces can be expressed as

3 $${\rm{Dir}}({\rm{\theta }},{\rm{\phi }})=\frac{4\pi {f}^{2}(\theta ,\phi )}{{\iint }_{\theta ,\phi }{f}^{2}(\theta ,\phi )sin\theta d\theta d\phi }$$

**Figure 4:**
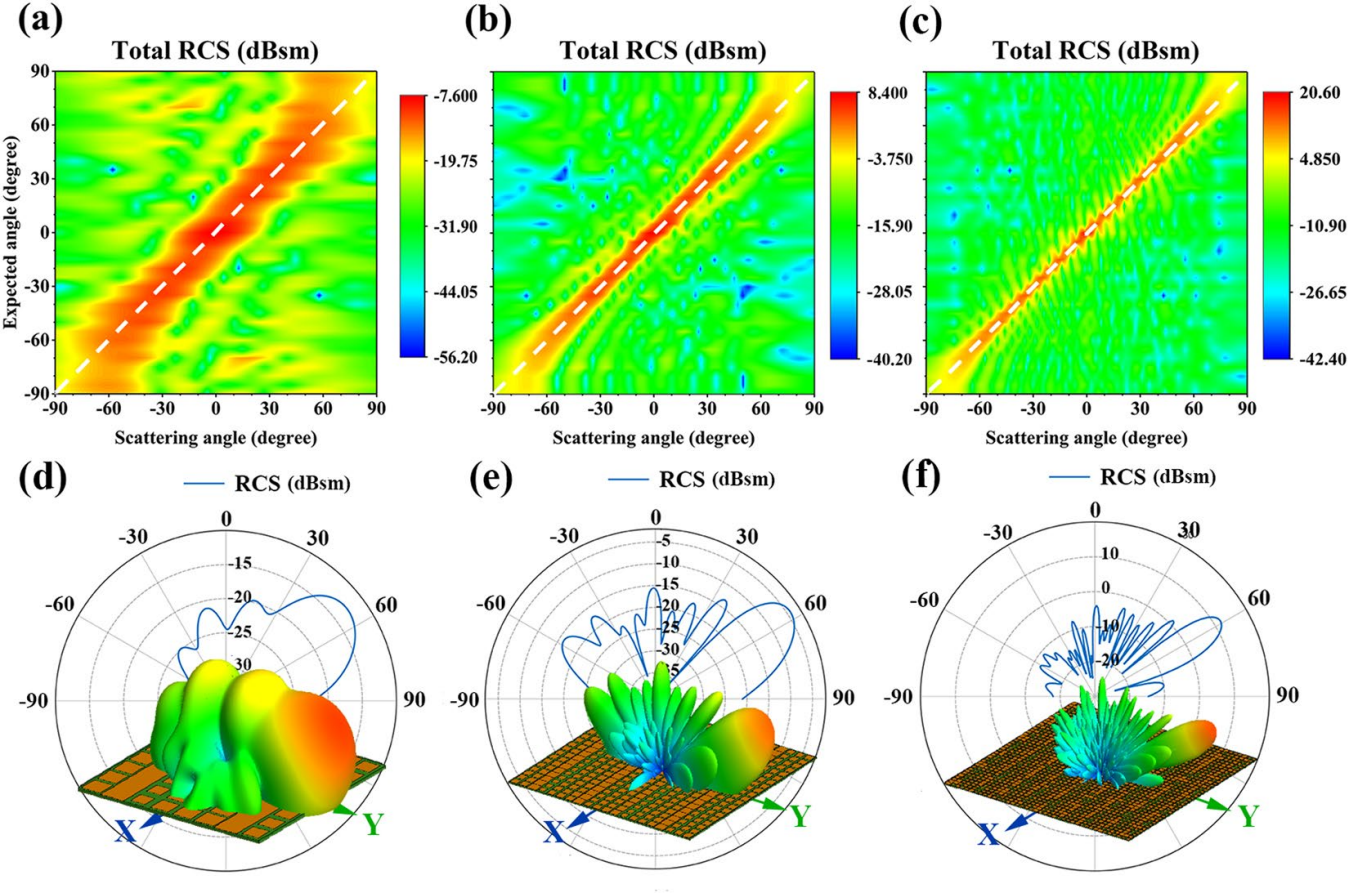
The simulation of far field RCS under normal incidence at 10 GHz in the YOZ plane. Figure 4a–c show the radiation angles *θ* of different optimal layouts versus their expected angles. The white dash lines represent the expectancy for (**a**) 8 × 8 arrays; (**b**) 20 × 20 arrays; (**c**) 40 × 40 arrays. Figure 4d–f describe the far field RCS of the optimized arrays in dB scale: (**d**) 8 × 8 arrays; (**e**) 20 × 20 arrays; (**f**) 40 × 40 arrays. The directions of the max radiation energy are *θ* = 53°, 57° and 59°, respectively. Their directivities are 31.51 dBi, 203.93 dBi and 715.53 dBi. The polar plots in Fig. 4d–f represent the far field RCS in YOZ plane.

The directivity shows the radiation energy concentrated in the direction of *θ* and *φ*. With the enhancing of wave modulating, the side lobes of radiation are decreasing, which means the high directivity. For single lobe radiation, the *θ* and *φ* of highest directivity represent the direction of the strongest radiation energy. Therefore, the expected single lobe radiation is tried to modulate to the highest directivity by optimized design method. In order to investigate wave modulating, single lobe metasurfaces with different radiation directions have been optimized at 10 GHz. The different OFs have been set at every 5 degrees of the elevation angle *θ* from −90° to 90°. For the 8 × 8 arrays, the simulation results of optimal layouts are shown in Fig. 4a. The white dash line in the diagram represents a perfect fit between the obtained and the expected radiation angles. It is noted that the radiation agrees well with the expectancy when the absolute value of *θ* is below 53°. However, when the absolute value of the expected angle exceeds 53°, the radiation angle is always near to 53° and not meets the expectancy. In order to improve the performance of wave modulating, the 20 × 20 and 40 × 40 arrays have been optimized and analyzed in the same way, as shown in Fig. 4b,c. Note that the maximum absolute values of radiation angle consistent with the expectancies are 71° and 80°, respectively. It concludes that the increase of array elements leads to the expansion of angle range of wave modulating. Similar to the phased array antenna, the total radiation is a summation of every element’s radiation. Hence, the larger quantity of elements improves the flexible of wave modulating. To further confirm this point, we have simulated the optimized 8 × 8, 20 × 20 and 40 × 40 arrays, which expected radiation angles are 60°. The 3D views of far filed RCS in dB are shown in Fig. 4d–f, which polar plot parts represent the YOZ plane far field RCS. It is noteworthy that, for the 8 × 8, 20 × 20 and 40 × 40 arrays, the directions of the max radiation energy are *θ* = 53°, 57° and 59°, respectively. Their directivities are 31.51 dBi, 203.93 dBi and 715.53 dBi. The aperture efficiency can *η*_*α*_ be expressed as:

4 $${\eta }_{\alpha }=\frac{{\lambda }^{2}{\rm{D}}}{4\pi \,{\rm{A}}}$$

where the *D* is directivity and *A* is physical area of array. Then the aperture efficiencies of the proposed 8 × 8, 20 × 20, and 40 × 40 arrays are 0.6269, 0.6491 and 0.5694. Obviously, the number of the array elements directly affects the beam synthesis, including direction and magnitude. With the growing of the amount of array elements, the radiation direction reaches the expectance better, and the magnitude is even stronger. The desired single lobe is increasing and the undesired side lobes are decreasing, as shown in Fig. 4d–f. It is because the suppressing of side lobes and edge radiation. The quantity of elements improves the wave adduction and weakens the intensity of side lobes. In addition, the edge elements of finite metasurfaces may induce the discontinuities of phase that lead to the edge radiation. For the 8 × 8, 20 × 20 and 40 × 40 arrays, the percentages of edge elements are 43.75%, 19% and 9.75%, respectively.

The efficiency and effectiveness for wave modulating are improved by GA with a large number of iterations. Effectiveness evaluates the degree that how much the manipulated radiation direction meets expected direction. Efficiency refers to the speed for providing the desired distribution.

As in the previous examples, large arrays are able to promote the wave control. However, the huge quantity of elements need for large arrays are difficult to be distributed by traditional way according to generalized Snell’s law. Then, it is important to improve the efficiency. Table 2 displays the parameters that affect the efficiency and effectiveness. The distance between radiation direction and expected direction can measures the efficiency. The consumed time and convergence generation reveal the effectiveness.

**Table 2:** The parameters that evaluating the efficiency and effectiveness for wave modulating.

Array	Expected direction	Optimized direction	Sequence length (bit)	Convergence generation	Consumed time (sec)
8 × 8	60°	53°	192	390	3.7
20 × 20	60°	57°	600	2637	116.2
40 × 40	60°	59°	4800	9434	2264.1

## Optimizing Design of Broadband 3-Lobes Radiation

**Figure 5:**
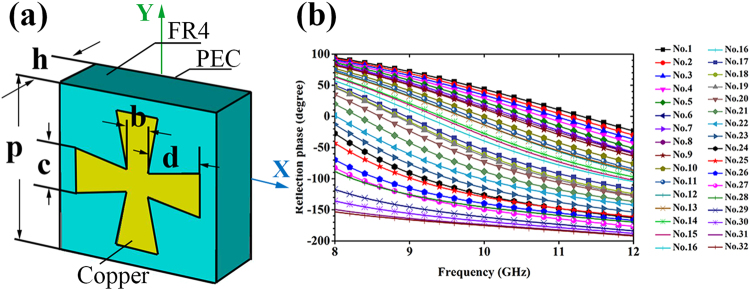
The unit diagram and the reflection properties of the broadband structure. (**a**) The parameters of cross-like patch are *b* = 1 mm with varying *c* and *d*. The FR4 substrate has a thickness of *h* = 3 mm, and the periodicity is *p* = 7.5 mm. (**b**) Reflection phase of different elements at X band obtained by FIT-simulation.

For the optimization of special radiation, the common 2-lobes or 4-lobes radiation is easily achieved through a checkerboard-like configuration^22,28^ because of its symmetry along the X axis and the Y axis, which is usually used in RCS reduction. Thus, the 3-lobes radiation without such symmetry is chosen here to confirm the validity of GA. In addition, bandwidth is another important factor that restricts the development of metasurfaces. For passive metasurfaces, it is necessary to explore elements with broadband phase gradient. Therefore, we choose thirty-two cross-like patterns as elements to construct the 20 × 20 arrays. The geometry and phase properties of elements at X band are shown in Fig. 5.

The curves of phase versus frequency are approximately parallel in Fig. 5b, which represents the broadband phase gradient. Note that the phase gradient at low frequency is larger than it at high frequency. Frequency dependence of far field performance is inevitable Table 3. In order to realize the wave manipulating in a relatively broadband, the OF should be rewritten as follow:

5 $$\begin{array}{rcl}{\beta }_{x} & = & |f({\theta }_{i},{\phi }_{i},{f}_{x})-f({\theta }_{i},{\phi }_{i},{f}_{0})|\\ O{F}_{{f}_{x}} & = & {\rm{\max }}[\sum _{i=1}^{N}-{\alpha }_{i}{\beta }_{x}\cdot f({\theta }_{i},{\phi }_{i},{f}_{x})]\end{array}$$

**Figure 6:**
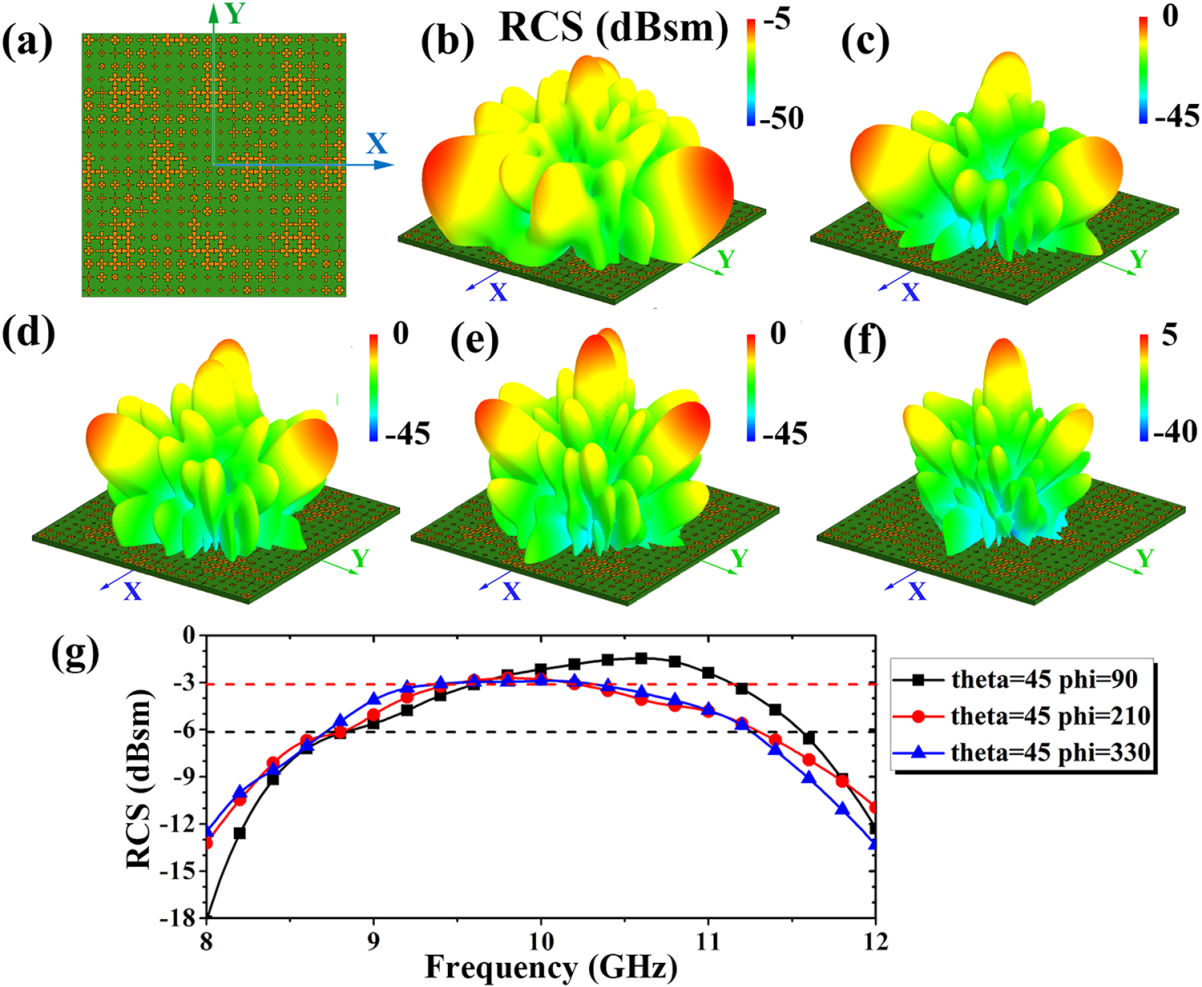
Geometry of optimization layout and the far field RCS simulated by MLFMA at X band in dB scale. (**a**) The size of the metasurfaces is 150 mm × 150 mm and the OF is *θ*_1_ = *θ*_2_ = *θ*_3_ = 45°, *φ*_1_ = 90°, *φ*_2_ = 210° and *φ*_3_ = 330° for three lobes of radiation. The simulation of the metasurface at X band are conducted at: (**b**) 8 GHz; (**c**) 9 GHz; (**d**) 10 GHz; (**e**) 11 GHz; (**f**) 12 GHz. (**g**) The RCS of three lobes versus the frequency.

Where the *β*_*x*_ is another broadband weight factor that measures the difference of the radiation far field function between center frequency *f*_0_ and other frequency *f*_*x*_. Note that we want to keep the *β*_*x*_ as small as possible, but the OF is the function of solving the maximum. Then, the *β*_*x*_ is turned into opposite number −*β*_*x*_. Summing all the −*β*_*x*_ of an optimized layout, the maximum is the best solution for the broadband case. In the case of 3-lobes optimization, it takes 143.2 seconds to reach the convergence. The simulation results of radiation at different frequencies are shown in Fig. 6. Obviously, the radiation fits the OF best at the center frequency 10 GHz. From 8.7 GHz to 11.3 GHz, the radiation nearly keeps stable with 3 dB difference away from the RCS of center frequency. The difference increases as the operating frequency shifting, which caused by the frequency-dependence of elements’ phase gradient.

**Table 3:** The pattern parameters of thirty-two elements.

No.	c (mm)	d (mm)	No.	c (mm)	d (mm)	No.	c (mm)	d (mm)
**1**	0	1	**12**	0	2.5	**23**	1.5	2.5
**2**	0.5	1	**13**	2	1.5	**24**	0.5	3
**3**	0	1.5	**14**	2.5	1.5	**25**	2	2.5
**4**	1.5	1	**15**	1	2	**26**	2.5	2.5
**5**	0.5	1.5	**16**	3	1.5	**27**	1	3
**6**	0	2	**17**	1.5	2	**28**	3	2.5
**7**	2.5	1	**18**	0.5	2.5	**29**	1.5	3
**8**	3	1	**19**	0	3	**30**	2	3
**9**	1	1.5	**20**	2	2	**31**	2.5	3
**10**	1.5	1.5	**21**	2.5	2	**32**	3	3
**11**	0.5	2	**22**	3	2

**Figure 7:**
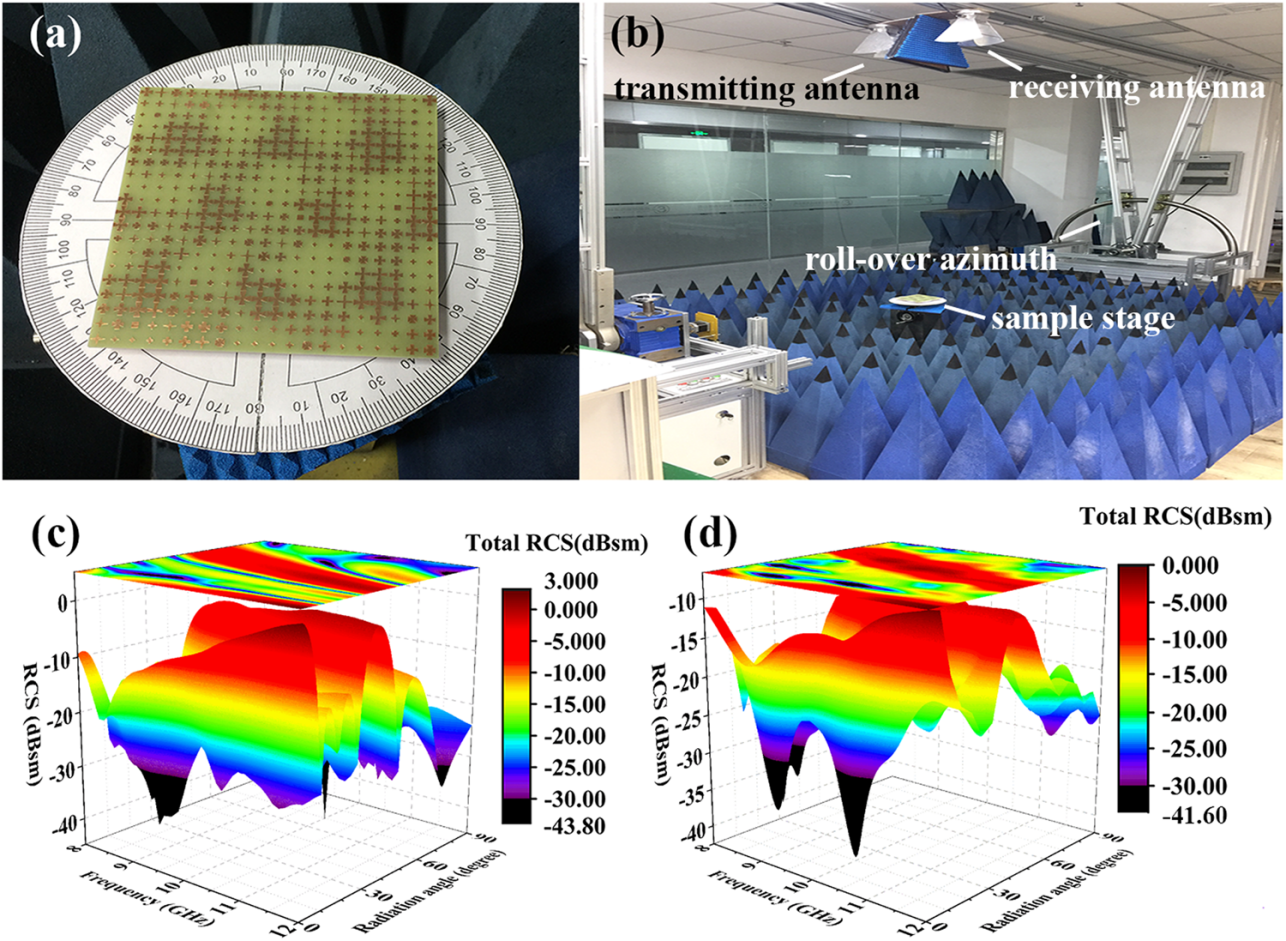
The fabricated metasurfaces, the measurement platform and the far field results. (**a**) The fabricated metasurface is place on the sample stage. (**b**) The setup of a bistatic RCS test platform. (**c**) The radiation image at *φ* = 90° has been simulated with varying angle *θ*. The horizontal axes represent incidence frequency and the angle of *θ*, respectively. The vertical axis depicts the RCS in dB. (**d**) The measured far field performance.

Radar cross section of the optimal metasurface has also been measured in a home-made microwave test platform. Figure 7a shows that the metasurface is placed on the sample stage. Two protractors are attached on the stage to ensure the location of the sample. Figure 7b displays the setup of a bistatic RCS test platform. It is done by using transmitting/receiving horn antennas with semicircular rail to adjust the elevation. A large number of pyramid absorbers surround the stage to eliminate the background reflection. Due to the limitation that the test platform has no roll-over azimuth, the transmitting/receiving antennas can only move in the YOZ plane, then only *θ* varies. Thus, the radiation pattern of metasurfaces in YOZ plane is measured at a specific X position, where one of the radiation lobes is located. The simulation and experiment results are shown in Fig. 7c,d. Generally, the two results are in good agreement and the wave manipulating of GA optimized layout is verified. In X band, the radiation direction is close to *θ* = 45°. With the increase of incidence frequency, the 3-lobes radiation is decreasing but the amplitude of normal reflection is increasing.

## Conclusion

In our works, the optimization method based on GA has been proved efficient and effectiveness for optimal layouts design of metasurfaces. The 8 × 8, 20 × 20 and 40 × 40 arrays with 8 basic elements have been proposed for single lobe radiation. Compared with the 8 × 8 arrays, the 20 × 20 and 40 × 40 arrays modulate wave more flexibly due to the large number of elements. With the increase of array elements, the effects of side lobes and edge radiation are weakened, leading to the high directivity. For broadband application, we fabricated the 20 × 20 layouts with 32 basic elements. By comparing the simulation and experimental results, it proves the relatively high efficiency of wave manipulating in X-band. Due to the frequency dependence of phase gradient in elements, the radiation at different frequencies is not easy to remain constant. In summary, the optimization method based on GA is adopted to design the layouts of metasurfaces for arbitrary wave manipulating, which is more efficient and smart than traditional ways. The GA promotes the design process and the results of optimal metasurfaces are in good agreement with the expectancy.
